# Unexpected deaths after endocrine surgery: learning from rare events using a national audit of surgical mortality

**DOI:** 10.1093/bjs/znac276

**Published:** 2022-08-05

**Authors:** Juanita N Chui, Alexander J Papachristos, Robert Mechera, Stan B Sidhu, Mark S Sywak, James C Lee, Justin Gundara, Christine Lai, Anthony R Glover

**Affiliations:** Endocrine Surgery Unit, Royal North Shore Hospital, Northern Sydney Local Health District and Northern Clinical School, Sydney Medical School, Faculty of Medicine and Health, University of Sydney, Sydney, New South Wales, Australia; Endocrine Surgery Unit, Royal North Shore Hospital, Northern Sydney Local Health District and Northern Clinical School, Sydney Medical School, Faculty of Medicine and Health, University of Sydney, Sydney, New South Wales, Australia; Endocrine Surgery Unit, Royal North Shore Hospital, Northern Sydney Local Health District and Northern Clinical School, Sydney Medical School, Faculty of Medicine and Health, University of Sydney, Sydney, New South Wales, Australia; Endocrine Surgery Unit, Royal North Shore Hospital, Northern Sydney Local Health District and Northern Clinical School, Sydney Medical School, Faculty of Medicine and Health, University of Sydney, Sydney, New South Wales, Australia; Endocrine Surgery Unit, Royal North Shore Hospital, Northern Sydney Local Health District and Northern Clinical School, Sydney Medical School, Faculty of Medicine and Health, University of Sydney, Sydney, New South Wales, Australia; Department of Surgery, Central Clinical School, Monash University, Melbourne, Victoria, Australia; Department of Surgery, Redland Hospital, Metro South and Faculty of Medicine, University of Queensland, Brisbane, Queensland, Australia; Department of Surgery, Logan Hospital, Metro South and School of Medicine and Dentistry, Griffith University, Logan, Australia; Division of Surgery, University of Adelaide, Adelaide, South Australia, Australia; Department of Surgery, Queen Elizabeth Hospital, Central Adelaide Local Health Network, Adelaide, South Australia, Australia; Endocrine Surgery Unit, Royal North Shore Hospital, Northern Sydney Local Health District and Northern Clinical School, Sydney Medical School, Faculty of Medicine and Health, University of Sydney, Sydney, New South Wales, Australia; Endocrine Cancer Program, Cancer Theme, Kinghorn Cancer Centre, Garvan Institute of Medical Research, St Vincent’s Clinical School. Faculty of Medicine, University of New South Wales, Sydney, New South Wales, Australia

## Abstract

**Background:**

The mortality rate is low in endocrine surgery, making it a difficult outcome to use for quality improvement in individual units. Lessons from population data sets are of value in improving outcomes. Data from the Australian and New Zealand Audit of Surgical Mortality (ANZASM) were used here to understand and elucidate potential systems issues that may contribute to preventable deaths.

**Methods:**

ANZASM data relating to 30-day mortality after thyroidectomy, parathyroidectomy, and adrenalectomy from 2009 to 2020 were reviewed. Mortality rates were calculated using billing data. Thematic analysis of independent assessor reports was conducted to produce a coding framework.

**Results:**

A total of 67 deaths were reported, with an estimated mortality rate of 0.03–0.07 per cent (38 for thyroidectomy (0.03–0.06 per cent), 16 for parathyroidectomy (0.03–0.06 per cent), 13 for adrenalectomy (0.15–0.33 per cent)). Twenty-seven deaths (40 per cent) were precipitated by clinically significant adverse events, and 18 (27 per cent) were judged to be preventable by independent ANZASM assessors. Recurrent themes included inadequate preoperative assessment, lack of anticipation of intraoperative pitfalls, and failure to recognize and effectively address postoperative complications. Several novel themes were reiterated, such as occult ischaemic heart disease associated with death after parathyroid surgery, unexpected intraoperative difficulties from adrenal metastasis, and complications due to anticoagulation therapy after thyroid surgery.

**Conclusion:**

This study represents a large-scale national report of deaths after endocrine surgery and provides insights into these rare events. Although the overall mortality rate is low, 27 per cent of deaths involved systems issues that were preventable following independent peer review.

## Teaser

This study represents the first large-scale national report of deaths after endocrine surgery, and provides insights into these rare events that can be used for education and future prevention strategies. Although the overall mortality rate is low, over one-third of deaths involved systems issues that were considered preventable following independent peer review. The findings provide insights into these rare events that may be used to inform future preventative practices and continuing professional development of surgeons.

## Introduction

Surgical audit is a core component of clinical practice, through which the quality and safety of surgical care is evaluated and compared with accepted standards. In Australia, the Royal Australasian College of Surgeons oversees a nationwide prospective audit of surgical deaths, with established peer-review and outcome-reporting pathways. The majority (over 98 per cent) of practising surgeons across public and private sectors participate in this audit^1^. The data collected present a unique opportunity to examine the current standard of care and reflect on more than a decade of surgical experience.

Mortality rates in endocrine surgery are exceptionally low, and so existing data describing such events are limited. To date, there has been no comprehensive review of factors associated with death after thyroid, parathyroid, and adrenal surgery. The Australian and New Zealand Audit of Surgical Mortality (ANZASM) has been used to examine trends in mortality across various surgical specialties, including neurosurgery^2^, cardiothoracic surgery^3^, urology^4,5^, and general surgery^6,7^. These studies have demonstrated that, by examining the small subset of operations resulting in patient death, potential gaps and pitfalls in clinical management may be identified to direct future improvement strategies in the quality and safety of surgical care.

By using large-volume longitudinal data, this study aimed to provide a description of deaths associated with endocrine surgery, and explore the preventable systems and process issues that may be associated with them. Lessons learned from these unexpected events could inform future practice and continuing professional development for surgeons.

## Methods

### ANZASM data and peer-review process

The ANZASM is an audit process that is independent, external, peer-reviewed, systematic, routine, objective and confidential, with the aim of gathering information on factors involved in the death of patients undergoing surgical treatment^8^. ANZASM is notified of all deaths that occur within 30 days of a surgical procedure by the medical records departments of hospitals in Australia, excluding deaths that occur in New South Wales (NSW), which are audited independently by the ANZASM-affiliated Collaborating Hospitals’ Audit of Surgical Mortality (CHASM). Data from both public and private hospital sectors are captured, and participation is mandatory for practising surgeons as part of continuing professional development^1^. The structure and methodology of the audit process^3^ are briefly summarized below.

Following notification of a death, a standardized surgical case form is completed by the treating surgeon^9^. The deidentified data are sent for first-line assessment by an independent surgeon within the same specialty at a different hospital. Should the first-line assessor judge the case summary to be sufficient and find no clinical management issues, no further investigation is undertaken. If issues are identified or further information is requested, an independent and deidentified second-line assessment involving a detailed case-note review is conducted. The perceived impact of the incident on the outcome is recorded and classified as ‘made no difference to outcome’, ‘may have contributed to death’ or ‘caused the death of a patient who would otherwise have been expected to survive’. In addition, a judgement regarding the preventability of the incident is made, classified as ‘definitely preventable’, ‘probably preventable’, ‘probably not preventable’ or ‘definitely not preventable’.

### Data retrieval

Following ethics approval from the University of Sydney and Northern Sydney Local Health District, data extraction was performed by ANZASM and CHASM data managers for the interval June 2009–2020. Cases were retrieved irrespective of the specialty of the treating surgeon. All cases were reviewed manually for accuracy and relevance.

### Estimation of volumes and mortality

The ANZASM and CHASM do not record procedural volume. Data from the Medicare Benefits Schedule^10^ were therefore used to estimate the number of thyroidectomies, parathyroidectomies, and adrenalectomies that were performed during the audit period to determine crude mortality rates. These Medicare data account only for procedures billed through the private sector. Therefore, these figures were subsequently adjusted to account for the estimated average private health insurance coverage, using data from the Australian Prudential Regulation Authority^11^.

### Qualitative analysis

Surgeon reports were analysed for all patients. Cases that underwent second-line review were additionally associated with narrative reports, further detailing complications and process issues identified by peer review. All available reports were analysed thematically using a reflexive approach, as described by Braun and Clarke^12^, to produce a coding framework^13^. Several narrative reports were initially reviewed independently and coded by two authors. The final coding framework was developed in consultation with the senior author, with discussion of discrepancies, although there was a high level of agreement among coders. All reports were then coded, and themes were constructed, mapped, and defined before the final reporting structure was agreed on. Within the coding framework, subthemes were explored and adverse events categorized as potentially preventable or non-preventable. The clinical vignettes in the results section are not direct excerpts from individual cases; rather, they summarize various case scenarios to highlight key themes while ensuring confidentiality.

## Results

**Fig. 1:**
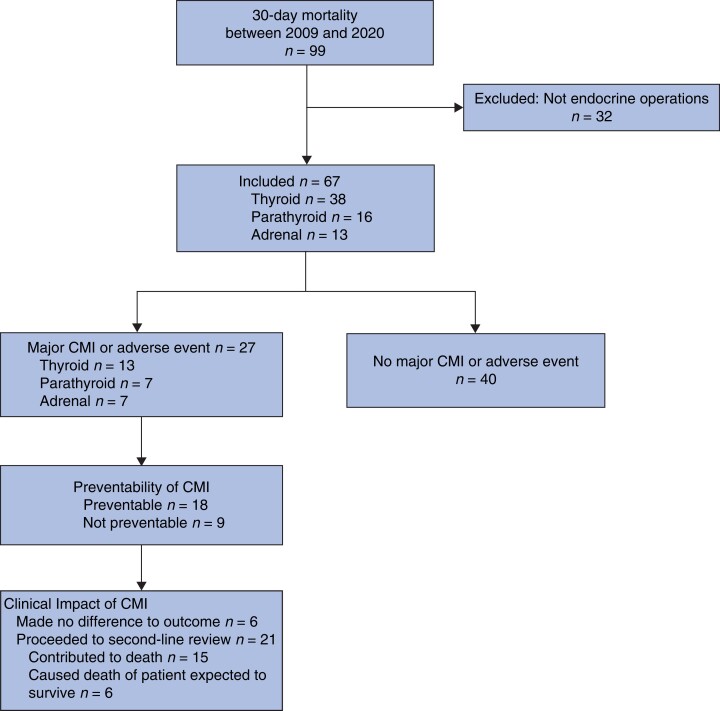
Flow diagram illustrating identification of 30-day mortality cases involving major clinical management issues or adverse events on peer review CMI, clinical management issue.

Data extraction from the ANZASM records 99 deaths with 30 days of operation with a keyword of thyroid, parathyroid or adrenal. Thirty-two were excluded because they were not associated with endocrine surgical procedures, leaving 67 for analysis. These included 38 deaths after thyroidectomy, 16 after parathyroidectomy, and 13 after adrenalectomy (*Fig. 1*).

### Overview of patient characteristics

Demographic characteristics are summarized in *Table 1*. The median age of the patients was 71 (range 32–96) years, and 28 (42 per cent) were men. The median duration of hospital stay was 10 (range 0–176) days; some patients had undergone an endocrine operation during a hospital stay for other reasons. For 63 patients (94 per cent), the primary operating surgeon was a consultant surgeon. Surgical and pathological details are shown in *Table 2*. Fifty operations were elective and 17 were unplanned. The latter were predominantly performed for a threatened airway (5), expedited management for a rapidly growing thyroid cancer after presentation with symptoms (4), calciphylaxis (3), or after inpatient evaluation and optimization of co-morbidities (3). Surgical postoperative complications occurred in 42 patients (63 per cent), the most common being postoperative haemorrhage (19 per cent) (*Table 3*). An unplanned return to theatre was needed for 16 patients (24 per cent).

**Table 1:** Patient characteristics

	No. of patients (n = 67)
**Age (years), median (range)**	71 (32–96)
**Sex**	
M	28 (42)
Not specified	3 (4)*
**Hospital type**	
Major public referral	21 (31)
Other public	11 (16)
Private	10 (15)
Not specified	25 (37)*
**Duration of hospital stay (days), median (range)**	10 (0–176)
**ASA fitness grade**	
I	3 (4)
II	11 (16)
III	30 (45)
IV	17 (25)
V	1 (1)
Not specified	5 (7)
**Co-morbidities**	
None	10 (15)
Cardiovascular	36 (54)
Respiratory	23 (34)
Renal	12 (18)
Diabetes	9 (13)
Advanced malignancy	17 (25)
Obesity	10 (15)
Neurological	8 (12)
Hepatic	3 (4)
Not specified	3 (4)*
**Risk of death**	
Expected	1 (2)
Considerable	7 (10)
Moderate	17 (25)
Small	13 (19)
Minimal	4 (6)
Not specified	25 (37)*

Values are *n* (%) unless otherwise indicated. *Not reported in Collaborating Hospitals’ Audit of Surgical Mortality.

**Table 2:** Procedure-specific mortality

	No. of patients
**Thyroid surgery**	38
Procedure	
Total or subtotal thyroidectomy	28 (74)
Hemithyroidectomy or lobectomy	8 (21)
Redo surgery	2 (5)
Abandoned (unresectable disease)	6 (16)
Surgical approach	
Open	37 (97)
Minimally invasive	1 (3)
Indication	
Malignancy	19 (50)
Papillary cancer	3 (8)
Follicular/Hürthle cell lesion	4 (10)
Medullary cancer	1 (3)
Anaplastic cancer	11 (29)
Graves’ disease	3 (8)
Toxic MNG/adenoma	2 (5)
Other	4 (11)
Not specified	10 (26)
**Parathyroid surgery**	16
Procedure	
Total or subtotal parathyroidectomy	11 (69)
Unilateral parathyroidectomy	3 (19)
Redo surgery	1 (6)
Not specified	1 (6)
Abandoned (AMI on induction)	1 (6)
Surgical approach	
Open	14 (88)
Minimally invasive	2 (13)
Indication	
Primary hyperparathyroidism	7 (44)
Secondary hyperparathyroidism	9 (56)
**Adrenal surgery**	13
Procedure	
Unilateral adrenalectomy	11 (85)
Bilateral adrenalectomy	2 (15)
Surgical approach	
Open	7 (54)
Laparoscopic transabdominal	3 (23)
Posterior retroperitoneoscopic	3 (23)
Indication	
Primary malignancy	4 (31)
Metastases	5 (38)
Cushing’s syndrome	3 (23)
Phaeochromocytoma	1 (8)
**Primary operating surgeon**	
Consultant	63 (94)
Fellow	1 (2)
Surgical trainee	1 (2)
Not specified	2 (3)

Values are *n* (%). MNG, multinodular goitre; AMI, acute myocardial infarction.

**Table 3:** Postoperative complications

	Total (*n* = 67 )	Thyroidectomy(*n* = 38)	Parathyroidectomy(*n* = 16)	Adrenalectomy(*n* = 13)
**Postoperative complications**	58 (83)	33 (87)	13 (81)	12 (92)
Postoperative haemorrhage	13 (19)	8 (21)	0 (0)	5 (38)
Clinically significant infection	10 (15)	5 (13)	3 (19)	2 (15)
Respiratory failure	10 (15)	9 (24)	1 (6)	0 (0)
Pulmonary embolism	4 (6)	2 (5)	0 (0)	2 (15)
Cardiac	16 (24)	7 (18)	8 (50)	1 (8)
Other	5 (7)	2 (5)	1 (6)	2 (15)
**ICU treatment**	48 (72)	29 (76)	14 (17)	5 (38)
**Unplanned return to theatre**	16 (24)	7 (18)	2 (13)	7 (54)

Values are *n* (%).

### Crude mortality rates

An estimated 98 120 procedures were performed in the audit period based on national Medicare data, including 67 453 thyroidectomies, 26 696 parathyroidectomies, and 3971 adrenalectomies. These figures were used to calculate the upper estimate of the mortality rate range, dividing the number of deaths by the total number of procedures performed in the public system. Allowing for an estimated 45.8 per cent rate of private health insurance coverage, the total number of procedures undertaken across public and private sectors was estimated. This estimate was used to calculate the lower limit of the mortality rate range. From these calculations, the estimated mortality rates were 0.03–0.06 per cent for thyroidectomy, 0.03–0.06 per cent for parathyroidectomy, and 0.15–0.33 per cent for adrenalectomy.

### Systems and process issues

On peer review, 27 deaths (40 per cent) were classified by assessors to be associated with clinical management issues (*Fig. 1*). Twenty-one of the 27 were associated with systems and process issues that had a significant impact on clinical outcome, that is they ‘may have contributed to death’ or ‘caused the death of a patient who would otherwise have been expected to survive’, and 18 of the 27 deaths were judged to have been preventable (*Table 4*). The 6 remaining clinical management issues were not felt to be associated with system and process issues, and the death was thought to be due to disease-related issues that were not preventable. For other non-preventable deaths, precipitating events included acute myocardial infarctions, cardiac failure, pulmonary embolism, and unrelated abdominal pathology that developed during the same admission after an endocrine surgical operation. These were not found to be preventable and/or related following peer review, with no clinical management issues identified.

**Table 4:** Australian and New Zealand Audit of Surgical Mortality peer review outcomes

	No. with clinically significance issues (*n* = 27)
**Clinical impact**	
Made no difference to outcome	6 (22)
May have contributed to death	15 (56)
Caused death of patient expected to survive	6 (22)
**Preventability**	
Definitely	6 (22)
Probably	12 (44)
Probably not	7 (26)
Definitely not	2 (7)

Values are *n* (%).

### Causes of death

In addition to the clinically significant adverse events identified by ANZASM peer review, potential systems and process issues identified on thematic analyses of all narrative reports obtained from the primary surgeon and peer reviewers were coded by the study authors into major themes relating to preoperative, intraoperative, and postoperative stages of care (*Table 5* and *Table S1*). In this analysis of the 67 deaths, 43 (64 per cent) were associated with themes related to potentially preventable adverse events (*Table 5*). To illustrate themes, representative clinical vignettes are presented, which combine elements from several reports, but do not describe individual patients so that confidentiality can be maintained.

**Table 5:** Major preventable themes and frequencies identified from analysis of narrative reports

	Total (*n* = 67)	Thyroidectomy (*n* = 38)	Parathyroidectomy (*n* = 16)	Adrenalectomy (*n* = 13)
**Preoperative**				
Preoperative assessment	16 (24)	8 (21)	7 (44)	1 (8)
Decision to operate	9 (13)	5 (13)	2 (13)	2 (15)
Choice of operative procedure	3 (4)	1 (3)	0 (0)	2 (15)
**Intraoperative**				
Delay in definitive treatment	2 (3)	0 (0)	1 (6)	1 (8)
Technical error	11 (16)	5 (13)	1 (6)	5 (38)
Intraoperative decision-making	4 (6)	1 (3)	0 (0)	3 (23)
**Postoperative**				
Delayed recognition of complications	5 (7)	3 (8)	1 (6)	1 (8)
Inappropriate management	7 (10)	6 (16)	0 (0)	1 (8)
Inadequate DVT prophylaxis	1 (1)	0 (0)	0 (0)	1 (8)
**Documentation and communication**	2 (3)	2 (5)	0 (0)	0 (0)
**No preventable theme identified**	24 (36)	15 (39)	6 (38)	3 (23)

Values are *n* (%). DVT, deep vein thrombosis.

### Preoperative issues

‘*A patient underwent an elective parathyroidectomy for secondary hyperparathyroidism in the context of end-stage renal failure. After surgery the patient went into cardiac arrest and, following initial resuscitation, an echocardiogram revealed profound cardiac dysfunction and severe valvular pathology, which had not been identified before surgery. The patient subsequently deteriorated and resuscitative efforts were withdrawn following consultation with the patient’s family.*’

Inadequate preoperative assessment was identified as the leading clinical management issue contributing to patient death in this study (16 of 67, 24 per cent), and was most notable in the context of parathyroid surgery (*Table 5*). Among 27 cases with clinical management issues identified on first- or second-line peer review, the potential benefit of surgery was questioned in 9 patients when weighed against the risk of adverse outcomes based on the medical and anaesthetic risk profile. Specific recommendations that arose from peer review included the need for more careful patient selection, focusing on preoperative decision-making, patient optimization, and surgical planning. Analysis demonstrated that, in the setting of end-stage renal failure, 9 deaths were associated with significant issues in preparation for surgery or the decision to operate, emphasizing the prevalence and severity of occult ischaemic heart disease in this group (*Table 5*). Failure to recognize or adequately address these co-morbidities during preoperative work-up led to a range of medical complications after surgery. Of note, there were no significant issues identified for patients who died after surgery for primary hyperparathyroidism.

Among deaths that occurred after thyroidectomy, 11 of 38 were related to anaplastic thyroid cancer, with death occurring within 30 days of surgery owing to local complications following failed attempts at resection (*Table 2*). Six operations were elective and 5 were performed as unplanned procedures because of concerns about airway compromise. In the majority of these patients, the histological diagnosis was not made before surgery, but the diagnosis was suspected based on the clinical presentation. Review of case reports outlined a rapidly enlarging neck mass with evolving airway compromise, with an inconclusive fine-needle aspiration result. In some patients, resection was attempted as the thyroid was judged to be resectable based on clinical examination and reassuring imaging features, and in others surgery was undertaken because of concerns regarding a threatened airway.

### Intraoperative issues

*‘A patient underwent a posterior retroperitoneoscopic adrenalectomy for an adrenal metastasis larger than 4 cm. The surgery was technically challenging, with a significant peritumoral desmoplastic reaction, resulting in bleeding from the inferior vena cava. After conversion to an anterior laparotomy, bleeding was controlled. However, the patient deteriorated, developing a combination of acidosis, coagulopathy, renal failure, and liver failure.’*

Death owing to intraoperative issues was described as a preventable theme for a total of 15 of 67 patients (22 per cent) (*Table 5*). Subthemes included delay in acquiring surgical assistance to deal with unexpected intraoperative findings, and the decision to continue with an operation at a critical juncture after complications had arisen. Death from intraoperative haemorrhage was reported predominantly in adrenalectomy for malignancy, and the distorted or aberrant anatomy associated with redo thyroid surgery.

Among deaths related to adrenalectomy, the choice of posterior retroperitoneoscopic adrenalectomy as the surgical approach was identified as a clinically significant issue by assessors in 2 of 13 patients. When undertaking posterior retroperitoneoscopic adrenalectomy, surgeons are faced with a relatively unfamiliar anatomical view and a small working space. Therefore, appropriate patient selection is important. Tumours larger than 5 cm, or malignant tumours that invade normal tissue planes, present added technical difficulty and were factors associated with mortality. Of note, in this study all resections of primary adrenal malignancies were performed using an open approach.

### Postoperative issues

*‘A patient underwent total thyroidectomy for thyrotoxicosis. In recovery, the patient complained of difficulty breathing and was reassured. The patient subsequently developed increasing respiratory distress and neck swelling on the ward. Upon further deterioration, the wound was opened, and the patient transferred back to the operating theatre, where they went into cardiac arrest. After significant delay, a surgical airway was established. The patient died shortly thereafter.’*

Postoperative issues associated with mortality were reported in 13 (19 per cent) of all 67 reported deaths, and most frequently observed after thyroidectomy (*Table 5*). Most deaths resulted from postoperative haemorrhage or respiratory complications. Deaths secondary to postoperative haemorrhage were only seen after total thyroidectomy; 1 death occurred owing to a delayed bleed (5 days after surgery), upon restarting anticoagulation. Of note, none of the deaths after parathyroidectomy and thyroidectomy were related to hypocalcaemia. All issues related to postoperative care were judged by the assessors to have been potentially preventable.

Complications associated with anticoagulant therapy were seen in 4 of 67 patients. For adrenal surgery, inappropriately withholding thromboprophylaxis after major abdominal surgery was identified as a contributing factor in 2 of 13 deaths. In the event of delayed haemorrhage after postoperative reanticoagulation, there were no mistakes identified in prescribing practices.

Recurrent systems issues identified in the postoperative phases included delayed recognition (7 per cent) and inappropriate management (10 per cent) of complications (*Table 5*). The importance of surgical leadership in the proactive management of complications, as well as in ensuring the appropriate level of postoperative monitoring, were major themes that emerged. Contributing factors included unclear postoperative instructions for nursing staff, lack of senior review, and suboptimal communication among involved teams. Three deaths involved respiratory failure, precipitated by early postoperative aspiration associated with recurrent laryngeal nerve injury. Intraoperative neuromonitoring was not used in these patients and, as no significant voice change was identified on the first postoperative day, there was a potential delay in diagnosis of the recurrent laryngeal nerve injury.

## Discussion

This study aimed to provide a description of deaths associated with endocrine surgery and to explore the preventable factors often associated with them. Overall, mortality rates of less than 0.06 per cent for thyroidectomy, below 0.06 per cent for parathyroidectomy, and under 0.33 per cent for adrenalectomy compare favourably with international data, with recent studies reporting mortality rates of 0.2 per cent for thyroidectomy^14–16^, 0.14–0.84 per cent for parathyroidectomy^17,18^, and 0.5–1 per cent following open or laparoscopic adrenalectomy^19,20^. Although these are crude estimates, they reinforce that death after endocrine surgery is a rare occurrence.

The results of this study, however, highlight that death after endocrine surgery is often associated with potentially preventable systems and process issues. Compared with the overall ANZASM figures for all surgical procedures in Australia^1^, mortality after endocrine surgery is more likely to be associated with preventable systems and process issues (27 *versus* 12 per cent). The profile of thyroidectomy-related deaths is consistent with previously published reports^14^, whereas the data describing deaths after parathyroidectomy and adrenalectomy are novel. These results emphasize the impact of inadequate preoperative assessment and optimization, errors in intraoperative decision-making, and failure to rescue in the setting of early postoperative complications, which lead to preventable deaths. Through thematic analysis of surgeon and auditor reports, this study has identified three important lessons that may help to prevent similar deaths from occurring in future, grouped into ‘when to operate’, ‘how to operate’, and ‘how to rescue’.

This study highlights the importance of thorough preoperative assessment. Most patients in this study were expected to survive according to surgeon and peer reviews, emphasizing the potential preventability of these deaths and the importance of ensuring adequate preoperative optimization. Of note, occult ischaemic heart disease and cardiorespiratory co-morbidities are common in patients with secondary hyperparathyroidism and must be evaluated specifically^21–23^, perhaps in the form of a standardized preoperative protocol.

In the context of anaplastic thyroid cancer, the preoperative histological diagnosis is critical and the extent of disease needs to be evaluated carefully^24^. As this is an aggressive and rare pathology, patients should be managed in specialist centres, with preoperative diagnosis allowing early referral and avoidance of partial resection attempts. Core biopsy is usually required for definitive diagnosis and should be performed in all patients, as it also allows molecular testing to identify targetable mutations. As with many rare pathologies, the association between surgeon and hospital volumes and improved clinical outcomes is well established^25–28^. The multidisciplinary team in a high-volume specialist centre will have a greater awareness of and access to clinical trials for these patients, facilitating the use of novel targeted treatments, which may result in a significant improvement in survival and downstaging of disease^29^. However, it must be acknowledged that these novel treatment paradigms have only evolved over the past few years, and so were not part of the clinical armamentarium for some of the present study interval^24^.

In relation to how to operate, careful surgical planning and anticipation of technical difficulty are essential in preventing intraoperative pitfalls. In this study, 5 of 13 deaths after adrenal surgery were associated with procedures performed for metastases. Adrenalectomy for metastatic disease carries additional technical challenges, with loss of the normal adventitial planes along major vascular structures. These cases emphasize the importance of an awareness of such pitfalls when deciding on the surgical approach. Appropriate patient selection for minimally invasive techniques is essential, particularly given the inherent difficulties associated with converting posterior retroperitoneoscopic adrenalectomy to an open operation. These findings may be increasingly important as systemic treatments improve and the indications for metastectomy expand^30–33^.

Valuable insights can be gained from cases of failure to rescue. In the setting of thyroidectomy, postoperative haematoma is potentially life-threatening owing the risk of airway compromise. Although a well recognized complication, deaths from post-thyroidectomy bleeds still occur^34^, despite clear postoperative instructions and established postoperative management protocols. These findings serve as a reminder that the clinical signs may be subtle and difficult to recognize, and that junior medical and ward staff require ongoing and regular education to ensure early recognition. Furthermore, they must appreciate the importance of timely and clear communication in escalating concerns during the early postoperative phase^35^. In addition to early recognition, timely management of complications is essential if the spiral of sequelae is to be prevented.

There is conflicting evidence regarding the impact of anticoagulant use on post-thyroidectomy bleeding^36,37^. In the era of sutureless thyroidectomy with advanced energy devices^38^, it appears that there is a small risk of delayed bleeding around 5–7 days after surgery in patients who are therapeutically anticoagulated. The risk should be considered during patient counselling at discharge, emphasizing the importance of urgent re-presentation in the event of neck swelling. If there is an unequivocal indication for restarting anticoagulation before 1 week after surgery, a traditional approach to control of the vascular pedicles could be considered.

In the setting of recurrent laryngeal nerve injury, there must be a high degree of vigilance for diagnosis and proactive management of respiratory complications. Permanent and temporary recurrent laryngeal nerve injuries are estimated to occur in 0.3–3 and 5–8 per cent of thyroidectomies respectively^39,40^, and both carry a significant risk of early postoperative morbidity. Three deaths identified in this audit were related to early postoperative aspiration pneumonia precipitated by recurrent laryngeal nerve injury after total thyroidectomy. Although intraoperative neuromonitoring does not prevent injury^41,42^, it allows immediate recognition of its occurrence. Early diagnosis may allow prevention of respiratory complications.

The importance of surgical leadership was noted in several of the case reports, and is an area that requires ongoing focus and development. This begins with anticipating technical difficulties and planning for all eventualities. It involves modelling effective communication among team members and maintaining situational awareness, especially in the emergency setting^43^. Complications are an inevitable part of surgery, but it is the ability to rescue that often ultimately determines a patient’s outcome. Accordingly, training in non-technical skills and team-based crisis-situation management may be of value in improving outcomes from preventable complications.

This study has several limitations. The ANZASM and CHASM do not record procedural volumes, so postoperative mortality rates were extrapolated crudely from national Medicare data and rates of private health insurance coverage. Moreover, the subjectivity of surgeon reports and the peer-review process must be acknowledged. Unless second-line assessment was conducted with full patient-note review, the details of patient deaths were provided by the treating surgeon, introducing the potential for bias. The subjectivity of assessments being completed by a single assessor must also be recognized. Similarly, the detail provided in surgeon reports was variable. This issue is, however, mitigated by the fact that, if there are any concerns regarding lack of information or potential management issues during the first-line review, a second-line in-depth assessment is mandatory. In addition, the ability of this study to use thematic analysis identified additional possible preventable themes that were not apparent to assessors reviewing individual cases. Finally, only procedures that resulted in death were explored in this study. In future studies, it may be of interest to examine cases that were rescued, as valuable insights may also be gained from reviewing cases in which adverse events occurred, but deaths were successfully prevented. Despite these issues, the present series provides insights into a rare group of adverse outcomes, with the hope of reducing similar events in future. Death after endocrine surgery is rare, but further research could explore the role of surgeon experience, training, surgical approaches, training in anatomy and embryology, new technology, and improving surgical leadership as possible targets for preventative strategies.

## Supplementary Material

znac276_Supplementary_Data
