# Supplementary material for: Unexpected deaths after endocrine surgery: learning from rare events using a national audit of surgical mortality
Source: Br J Surg. 2022 Aug 5;109(11):1164–71. doi: 10.1093/bjs/znac276 (PMC10364700; doi:10.1093/bjs/znac276)
Supplement: znac276_Supplementary_Data [file znac276_supplementary_data.docx]

**Table S1.** Summary of major themes from thematic analysis of narrative reports that may contribute to postoperative mortality

| Major themes | Subthemes | High risk group |
| --- | --- | --- |
| Preoperative | - Thorough preoperative assessment - Adequate medical optimisation, anticipating common occult comorbidities | Parathyroidectomy   - Careful evaluation of risk-benefit in the surgical management of secondary hyperparathyroidism. - Occult cardiovascular disease   Thyroidectomy   - Appropriate recognition and multidisciplinary management of anaplastic thyroid carcinoma |
| Intraoperative | - Adequate training to ensure the provision of high-quality endocrine surgery. - Optimize strategies for risk management and decision-making. - Anticipation and preparation for intraoperative technical difficulties and potential complications - Exercising vigilance and early involvement of subspecialty colleagues. | Adrenalectomy   - Advanced malignancy or metastatic disease. Distortion of anatomy and loss of the normal adventitial planes along major vascular structures.   Redo surgery   - Operating on recurrent pathology in a scarred field, with distorted anatomical planes. |
| Post operative | - Early recognition and aggressive management of postoperative complications - Appropriate level of postoperative monitoring and adequate senior review - Continuing education of ward staff and empowerment to escalate. | Thyroidectomy   - Post-operative haematoma and delayed bleeding risk in anticoagulated patients - Respiratory complications in setting of recurrent laryngeal nerve injury |
